# Imaging the TGFβ type I receptor in pulmonary arterial hypertension

**DOI:** 10.1186/s13550-023-00966-7

**Published:** 2023-03-22

**Authors:** Lonneke Rotteveel, Alex J. Poot, Esther J. M. Kooijman, Robert C. Schuit, Ingrid Schalij, Xiaoqing Sun, Kondababu Kurakula, Chris Happé, Wissam Beaino, Peter ten Dijke, Adriaan A. Lammertsma, Harm Jan Bogaard, Albert D. Windhorst

**Affiliations:** 1 Department Radiology and Nuclear Medicine(s), (Amsterdam Cardiovascular Sciences), Amsterdam UMC, VU University Medical Center, de Boelelaan 1117, Amsterdam, The Netherlands; 2 Department Pulmonary Medicine, (Amsterdam Cardiovascular Sciences), Amsterdam UMC, VU University Medical Center, de Boelelaan 1117, Amsterdam, The Netherlands; 3 Department Cell and Chemical Biology, Leiden University Medical Center, Einthovenweg 20, Leiden, The Netherlands; 4 Oncode Institute and Leiden University Medical Center, Einthovenweg 20, Leiden, The Netherlands

**Keywords:** TGFβ type I receptor, ALK5, Positron emission tomography, Pulmonary arterial hypertension, SuHx, MCT, Carbon-11, Fluorine-18

## Abstract

Transforming growth factor β (TGFβ) activity is perturbed in remodelled pulmonary vasculature of patients with pulmonary arterial hypertension (PAH), cancer, vascular diseases and developmental disorders. Inhibition of TGFβ, which signals via activin receptor-like kinase 5 (ALK5), prevents progression and development of experimental PAH. The purpose of this study was to assess two ALK5 targeting positron emission tomography (PET) tracers ([^11^C]LR111 and [^18^F]EW-7197) for imaging ALK5 in monocrotaline (MCT)- and Sugen/hypoxia (SuHx)-induced PAH. Both tracers were subjected to extensive in vitro and in vivo studies. [^11^C]LR111 showed the highest metabolic stability, as 46 ± 2% of intact tracer was still present in rat blood plasma after 60 min. In autoradiography experiments, [^11^C]LR111 showed high ALK5 binding in vitro compared with controls, 3.2 and 1.5 times higher in SuHx and MCT, respectively. In addition, its binding could be blocked by SB431542, an adenosine triphosphate competitive ALK5 kinase inhibitor. However, [^18^F]EW-7197 showed the best in vivo results. 15 min after injection, uptake was 2.5 and 1.4 times higher in the SuHx and MCT lungs, compared with controls. Therefore, [^18^F]EW-7197 is a promising PET tracer for ALK5 imaging in PAH.

**Supplementary Information:**

The online version contains supplementary material available at 10.1186/s13550-023-00966-7.

## Introduction

Pulmonary arterial hypertension (PAH) is a condition in which pulmonary arterial obstruction increases vascular resistance, ultimately leading to right ventricular failure [1, 2]. To date, the diagnosis of PAH is based on invasive pressure measurements. As no diagnosis at the tissue level is made, little is known about the underlying pathophysiology of PAH in individual patients. Specific tissue biomarkers that target specific pathways involved in PAH have been contemplated, but are not available at present.

One of the targets involved in PAH is the cytokine transforming growth factor β (TGFβ). This cytokine signals via the TGFβ type I receptor, also known as activin receptor-like kinase 5 (ALK5). Preclinical PAH studies in Sugen 5416/hypoxia (SuHx) and monocrotaline (MCT) exposed rats demonstrated that inhibition of TGFβ signalling via ALK5 prevents the progression and development of PAH [3–5]. Activation of the TGFβ/ALK5 pathway has also been observed in pulmonary cells of remodelled pulmonary arteries of patients with idiopathic PAH [6]. This indicates that components of the TGFβ/ALK5 signalling pathway may provide potential biomarkers for early diagnosis and therapeutic evaluation of PAH. Positron emission tomography (PET) is a powerful technique to visualize and quantify cellular targets and molecular processes in vivo. PET is used in drug development, clinical research and patient care [7]. To date, assessing glucose uptake in the lungs using [^18^F]-2-fluoro-2-deoxy-D-glucose ([^18^F]FDG) is the only potential PET method for detecting PAH. The clinical relevance of [^18^F]FDG PET in PAH is, however, questionable, as its variable uptake does not correlate with the severity of the disease or survival, nor with the pathobiology of PAH [8, 9]. In contrast, ALK5 has been correlated with the pathobiology of PAH and uptake of ALK5 PET tracers should better correspond with the severity of the disease [3, 4, 6]. Therefore, preclinical PET imaging of ALK5 in well-established PAH models is the first step in developing an early non-invasive diagnostic tool for understanding the pathophysiology of PAH.

**Fig. 1:**
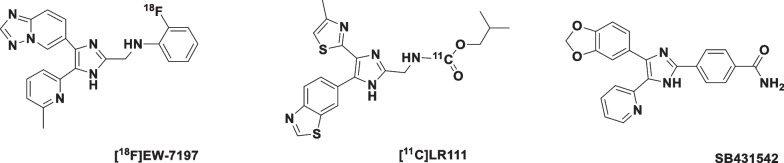
Molecular structures of the ALK5 targeting PET tracers, [^18^F]EW-7197 and [^11^C]LR111, and the selective ALK5 kinase inhibitor SB431542

To study PAH in a preclinical setting, commonly the monocrotaline (MCT) rat model is used [10]. MCT is metabolized in the liver by cytochrome P_450_-3A (CyP3A) into the active bifunctional cross-linking MCT pyrrole leading to vascular injury [10]. MCT mimics many features of human PAH, like swelling of mitochondria, generation of reactive oxygen species and peripheral extension of smooth muscle cells to small and large pulmonary arteries [11]. It should, however, be noted that there is no formation of obstructive intimal lesions in the peripheral pulmonary arteries of the rats [10]. A second model is the Sugen/hypoxia model, where Sugen 5416 (SU-5416), a VEGF inhibitor, together with chronic hypoxia exposure causes severe irreversible PAH associated with precapillary arterial endothelial proliferation, thereby representing a more realistic PAH model that resembles human disease [10]. The purpose of the present study was to investigate [^18^F]EW-7197 and [^11^C]LR111 (Fig. 1) as potential tracers for visualizing ALK5 in both animal models. Both [^18^F]EW-7197 and [^11^C]LR111 have previously been evaluated preclinically as potential ALK5 targeting PET tracers in MDA-MB-231 tumour xenografts [12].

## Materials and methods

### Immunofluorescence

Paraffin-embedded lung sections from control, MCT and SuHx rats were fixed and stained as previously described by Duim et al. [13]. Paraffin sections were deparaffinized and rehydrated. The sections were fixed in cold acetone (− 20 °C) for 1 min, air-dried for 1 h and hydrated in PBS. All sections were boiled for 40 min in Vector® Antigen Unmasking Solution (Vector) using a pressure cooker. After blocking with 1% BSA in 0.1% Tween-PBS, sections were incubated overnight at 4 °C with primary antibodies directed against ALK5 (1:1000 dilution; SantaCruz; Cat#sc-398), Pecam-1 (M-20; 1:1000 dilution; SantaCruz; Cat#sc-1506) and alpha smooth muscle actin (1:10,000 dilution; Aldrich, Cat#A2547). All sections were mounted with ProLong® Gold antifade reagent (Invitrogen, Carlsbad, USA) containing DAPI. For fluorescence quantification and comparison, sections were stained in a single run and images were collected in a single session with the same exposure time between different areas and different slides. Images were analysed using Caseviewer 2.3 software.

### Western blotting

Frozen lung tissues from controls, MCT and SuHx rats were homogenized, and cell lysates were prepared with NP-40 lysis buffer, and Western blotting was performed as described previously [14]. The protein suspensions were separated on a 10% gel, transferred to a nitrocellulose membrane, and incubated with blocking buffer (5% non-fat milk in PBS with 0.1% Tween 20). The blots were developed with antibodies for ALK5 (1:1000 dilution; SantaCruz; Cat#sc-398), vinculin (Sigma-Aldrich, Cat#V9131) or β-actin (Sigma-Aldrich, Cat# A5441) at 4 °C overnight, followed by horseradish peroxidase-conjugated antimouse or anti-rabbit (GE Healthcare) secondary antibodies using the Clarity ECL Substrate (1705061; Bio-Rad, Hercules, USA) and followed by scanning using light sensitive new RX medical 20 × 40 CM films (Fuji, Tokyo, Japan).

### Quantitative polymerase chain reaction (qPCR)

The frozen lung tissue was homogenized and total RNA was isolated using Tripure RNA isolation kit (Roche, Basel, Switzerland Cat#11667165001). cDNA synthesis was performed with iScript (Bio-Rad, Hercules, USA, Cat#170-8891), followed by real-time PCR using the SYBR Green and a Bio-Rad CFX Connect device. Primers used for real-time PCR for ALK5: Fw: GCTGACATCTATGCAATGGGCTTA, Rv: AGGCAACTGGTAGTCTTCGTGGA; GAPDH: Fw: GGTGGACCTCATGGCCTACA, Rv: CTCTCTTGCTCTCAGTATCCTTGCT. GAPDH served as a control for the amount of cDNA present in each sample. Data were analysed using the comparative difference in cycle number (ΔCT) method according to the manufacturer’s instructions. Q-PCR relative quantification was realized by calculating the ratio of the protein expression of interest over the GAPDH mRNA expression [15].

### Autoradiography

Sections of 10 µm thickness were cut using a Cryostat (Leica CM3050 S) and thaw mounted on Thermo Scientific Superfrost plus adhesion slides. The sections were stored at– 20 °C until further handling. Tissue sections were defrosted, washed three times for 5 min with 5 mM Tris HCl buffer, pH 7.4 and dried in a cold stream of air. The sections were incubated for 30 min with 0.5 mL of 5 mM Tris HCl buffer, pH 7.4, containing 1.0 MBq·mL^−1^ (6 ± 0 nM) [^11^C]LR111 tracer or 0.5 MBq mL^−1^ (7 ± 2 nM) [^18^F]EW-7197 with or without SB431542 (94 µM; Sigma-Aldrich, Zwijndrecht, The Netherlands) [16]. The sections were washed in cold 5 mM Tris HCl buffer, pH 7.4, (three times 1 min) followed by a dip in ice cold water. The sections were dried under an air stream and exposed to a phosphor storage screen (GE Healthcare lifescience, Buckinghamshire, UK) for 10 min. Screens were developed on a Typhoon FLA 7000 phosphor imager (GE Healthcare lifescience, Buckinghamshire, UK) and analysed using Image Quant TL v8.1.0.0 (GE Healthcare lifescience, Buckinghamshire, UK). Results were expressed in percentage and the healthy lungs were normalized to 100%. Statistical analysis was performed using Graphpad PRISM (v 5.02, Graphpad Software Inc). Tracer uptake in the tissue was compared with tracer uptake co-administered with SB431542 by using a one-tailed unpaired t-test. Differences were considered significant if *p* < 0.05. Adjacent tissue sections were used for autoradiography experiments.

### Animal models

Animal experiments were performed in accordance with the European Community Council Directive (2010/63/EU) for laboratory animal care and the Dutch Law on animal experimentation. The experimental protocol was validated and approved by the central committee for animal experimentation (CCD) and the local committee on animal experimentation of the Amsterdam University Medical Center, location VUmc. Animals were housed in groups under standard conditions (20–24 °C, 50–70% relative humidity, 12 h light/dark cycles) with a maximum of 6 animals per cage unless otherwise stated. In addition, they were provided with nesting material, sawdust, tap water and food (Teklad Global 16% Protein Rodent Diet, Harlan, Madison, WI, USA) ad libitum. Biodistribution studies (*n* = 32) were carried out in healthy male Wistar rats (214 ± 12 g, Envigo, Horst, The Netherlands). For PET studies, ten male Wistar rats (188 ± 20 g, Envigo, Horst, The Netherlands) were allocated to two different groups, a control group (*n* = 4) and an MCT-exposed group (*n* = 6) and Six Sprague Dawley (S.D.) rats (Charles River, Germany, 198 ± 6 g) were used as third group. MCT (Sigma-Aldrich, Zwijndrecht, The Netherlands) was administered subcutaneously to the Wistar rats as a single injection (60 mg kg^−1^) [17]. MCT rats were housed for two weeks after MCT injection. The S.D. rats were injected with Sugen (SU-5416, Tocris Bioscience, Bristol, UK) [18, 19] subcutaneously as a single injection (25 mg kg^−1^). SuHx rats were housed for 4 weeks after the Sugen injection in 10% oxygen, maintained by a nitrogen generator.

### Radiochemistry

The tracers were synthesized as reported previously [12]. Briefly, precursor for [^18^F]EW-7197 was synthesized according to Jin et al. [20]. [^18^F]EW-7197 was synthesized with a reductive amination reaction that was based on a method published by Vasdev et al. [21, 22]. Precursor for [^11^C]LR111 and LR111 was synthesized according to Amada et al. [23]. [^11^C]LR111 was synthesized using the so-called [^11^C]CO_2_ fixation method [24, 25].

### Biodistribution study and metabolite analysis in rats

A biodistribution study and metabolite analysis was carried out in healthy male Wistar rats (214 ± 15 g, Charles River, Germany). Rats were injected with 21 ± 15 MBq of [^11^C]LR111 in the tail vein under isoflurane anaesthesia (2% in O_2_ at 1 L min^−1^). Rats were sacrificed under similar isoflurane anaesthesia by exsanguination and dissected at 5, 15, 30 and 60 min post injection (p.i.) (*n* = 4 per time point). Rats were injected with 14 ± 5 MBq [^18^F]EW-7197 in the tail vein under isoflurane anaesthesia (2% in O_2_ at 1 L min^−1^). Rats were sacrificed under similar isoflurane anaesthesia by exsanguination and dissected at 5, 15, 30 and 45 min post injection (p.i.) (*n* = 4 per time point). Blood, heart, lungs, liver, kidneys, urine, bone and brain were collected, weighed and counted for radioactivity (Wallac 1210 Compugamma, PerkinElmer, Waltham, MA, USA). Biodistribution data were expressed as percentage injected dose per gram tissue (%ID·g^−1^) ± standard error of the mean (SEM). Blood at 15 min and 60 min p.i. ([^11^C]LR111) and blood at 15 min and 45 min p.i. ([^18^F]EW-7197), withdrawn from the abdominal aorta, (~ 7 mL) was collected in a heparin coated tube and centrifuged at 5000 rpm to separate plasma from blood cells (Hettich universal 32, Andreas Hettich GmbH & Co. KG, Tuttlingen, Germany). Solid phase extraction (SPE) cartridges (tC2, Waters, Ettenleur, The Netherlands) were preconditioned by washing with 6 mL MeOH and 2 × 6 mL H_2_O. Plasma (1 mL) was mixed with 6.0 M HCl (50 µL) and loaded on the SPE cartridge (> 99% recovery). The polar metabolite fraction was obtained by eluting the SPE cartridge with H_2_O (3 mL), the non-polar fraction by subsequent elution with MeOH (2 mL) and H_2_O (1 mL). Fractions were counted for radioactivity (Wallac 1210 Compugamma, PerkinElmer, Waltham, MA, USA). The percentage of intact tracer in the non-polar fraction was determined by online HPLC analysis on a Dionex (Sunnyvale, CA, USA) UltiMate 3000 HPLC equipment with Chromeleon software (version 6.8) equipped with a Gemini C18 column (5 μm, 10 × 250 mm, Phenomenex, Torrance, CA, USA) with a mixture of MeCN (A) and 0.1% TFA in water (B) as eluent according to the following scheme 0 min: 90% B; 12 min 15% B; 8 min 90% B at 3.5 mL.min^−1^ [^11^C]LR111 Rt = 10.7 min and [^18^F]EW-7197 Rt = 9.2 min. Results were expressed as percentage of intact tracer, polar metabolites and non-polar metabolites ± SEM (*n* = 4 for each time point).

### Echocardiography and haemodynamics

On the first day of the scanning sessions, all animals underwent echocardiographic assessments to measure right ventricular wall thickness (RVWT), right ventricular end diastolic diameter (RVEDD), tricuspid annular plane systolic excursion (TAPSE), cardiac output (CO) and estimated right ventricular systolic pressure (eRVSP), as published previously [26].

### PET imaging

**Fig. 2:**
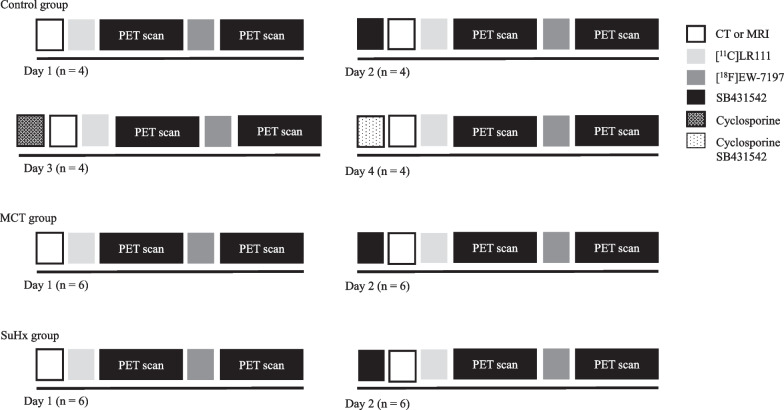
Experimental set-up of the PET scanning study

Dynamic PET imaging was performed with NanoPET/CT and nanoPET/MR 1 T scanners (Mediso Ltd., Hungary, Budapest) with identical PET components [27]. All injections were performed via a tail vein catheter. Rats were anesthetized with 4 and 2% isoflurane in 1 L·mL^−1^ oxygen for induction and maintenance, respectively. Rats were positioned on the scanner bed and the respiratory rate was monitored for the duration of the experiment, adjusting anaesthesia when required. Animals first underwent a computed tomography (CT) or magnetic resonance imaging (MRI) scan to keep the isoflurane exposure as low as possible (see Fig. 2 for the set-up of the experiment). All tracer injections were performed via a tail vein catheter using 4 and 2% isoflurane in 1 L min^−1^ oxygen for induction and maintenance, respectively (see Table 1 for animal weights and injected doses). Dynamic PET scans, starting at the time of tracer administration, were acquired for 60 min. Immediately after the [^11^C]LR111 scan, a bolus injection of [^18^F]EW-7197 was administered, in order to minimize the time that rats were exposed to isoflurane, and another dynamic PET scan was acquired. All animals received a baseline scan with [^11^C]LR111 and [^18^F]EW-7197 at day 1. At day 2, all animals received SB431542 30 min prior the injection of [^11^C]LR111. In addition, the control group received a cyclosporine A (Sigma-Aldrich, Zwijndrecht, The Netherlands) injection at day 3, 30 min prior to the injection of [^11^C]LR111. Finally, at day 4, SB431542 and cyclosporine A injections were administered 30 min prior to the injection of [^11^C]LR111. After these scanning experiments, the animals were sacrificed by cervical dislocation. Lung tissues were removed from MCT (*n* = 3), SuHx (*n* = 3) and healthy rats (*n* = 3). They were filled with 50% Tissue-Tek (VWR, Amsterdam, The Netherlands) and 50% physiological salt and snap frozen in liquid nitrogen. Positron emission scans were acquired in list mode and rebinned into the following frame sequence: 4 × 5, 4 × 10, 2 × 30, 3 × 60, 2 × 300, 1 × 600, 1 × 900 and 1 × 1200 s. The scans were reconstructed with a spatial resolution of 0.4 mm and were corrected for attenuation and scatter. The scans of the first 30 s were combined to correct for injection speed differences. Images were analysed using the freely available software AMIDE, version 1.0.4 (http://amide.sourceforge.net/). Regions of interest were drawn with a diameter of at least 4 mm in the lungs remote from the liver and heart, in order to avoid spill-over from these organs. Uptake was expressed as standard uptake values (SUV), which was calculated according to the following equation:

$${\text{SUV}} = \left[ {\% {\text{ uptake of the total tracer dose}}/{\text{volume of the region of interest}}} \right]/{\text{bodyweight}}$$

**Table 1:** Injected doses of [^18^F]EW-7197, [^11^C]LR111, SB431542, cyclosporine and animal weights of each experimental group

	Control group (*n* = 4)				MCT group (*n* = 6)		SuHx group (*n* = 6)	
	Day 1	Day 2	Day 3	Day 4	Day 1	Day 2	Day 1	Day 2
Animal weight (g)	289 ± 2				253 ± 20		301 ± 22	
[^11^C]LR111 (MBq)	12 ± 7	23 ± 0	16 ± 3	14 ± 5	17 ± 3	22 ± 4	22 ± 2	18 ± 1
[^18^F]EW-7197 (MBq)	14 ± 2	17 ± 5	13 ± 1	14 ± 1	15 ± 2	19 ± 4	18 ± 8	19 ± 1
SB431542 (mg kg^−1^)		20		20		20		20
Cyclosporine A (mg kg^−1^)			50	50				

Error bars indicate standard deviation. Statistical analysis was performed using Graphpad PRISM (v 5.02, Graphpad Software Inc). Lung uptake in the control group was compared with uptake in the diseased animals with a one-tailed unpaired t-test. Differences were considered significant if *p* < 0.05. The representative PET images were generated using VivoQuant2020.

## Results

### ALK5 expression in SuHx and MCT rat lungs

**Fig. 3:**
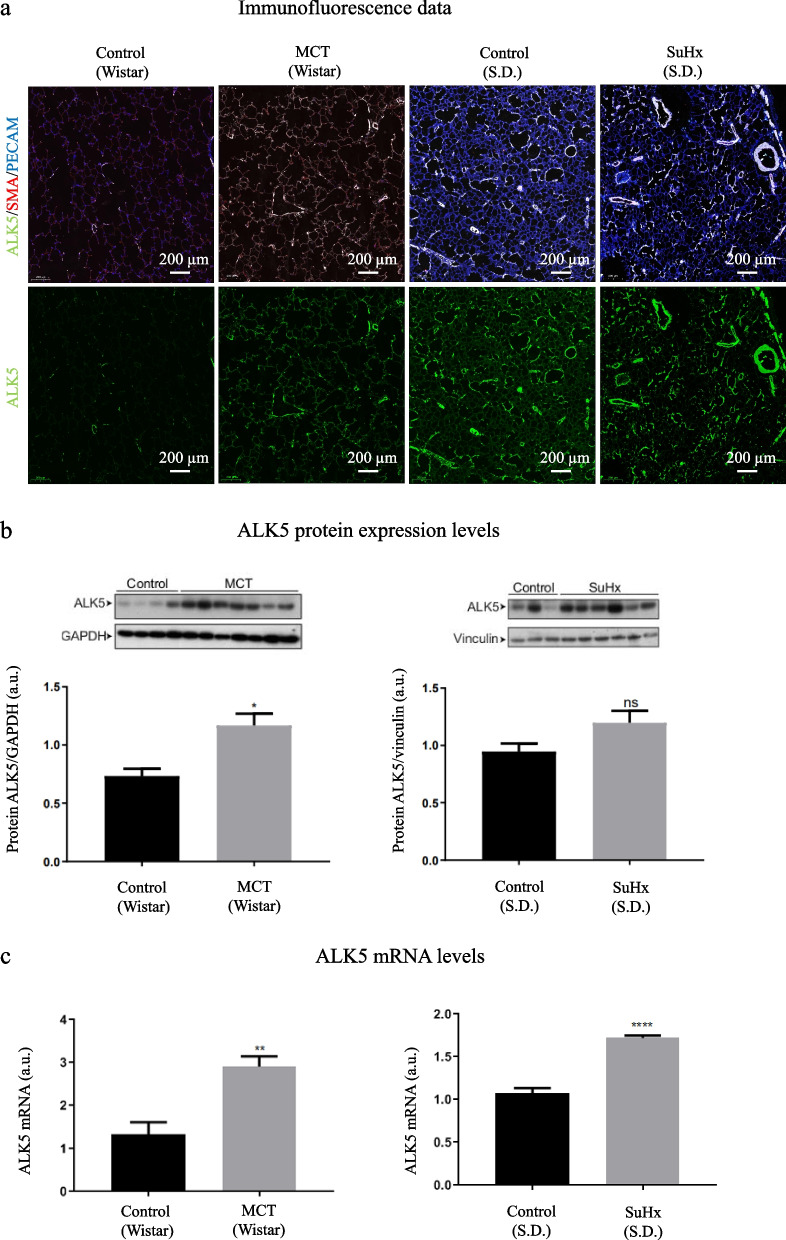
ALK5 expression in control, MCT-exposed (Wistar) and SuHx-exposed (S.D.) rat lungs: **a** immunofluorescence; **b** protein expression levels of ALK5 based on Western blot analysis. The western blot gel is cropped to improve the clarity of the image. See the Additional file 1 (Fig. 1) for the uncropped image; **c** mRNA levels of ALK5. ** represents a significant difference with *p* < 0.01, **** a significant difference with *p* < 0.0001

Immunofluorescence data clearly displayed increased ALK5 expression in diseased (SuHx- and MCT-exposed) lungs compared with lungs of control rats (Wistar and S.D.). In addition, SuHx-exposed lungs showed higher ALK5 expression than MCT-exposed lungs (Fig. 3a). Baseline expression of ALK5 in healthy Wistar rats was lower than in healthy S.D. rats (Fig. 3a). Western blot data showed the same trend as the immunofluorescence data (Fig. 3b). Consistent with these findings, the *m*RNA levels of ALK5 were significantly higher in both MCT and SuHx-induced PAH rat lungs (Fig. 3c).

### Radiochemistry

[^18^F]EW-7197 was obtained in a yield of 9 ± 5% decay corrected (*n* = 17), a molar activity of 149 ± 120 GB µmol^−1^ and a radiochemical purity > 95%. [^11^C]LR111 was obtained in a yield of 15 ± 5% decay corrected (*n* = 13), a molar activity of 143 ± 88 GBq µmol^−1^ and a purity > 95%.

### Autoradiography experiments

**Fig. 4:**
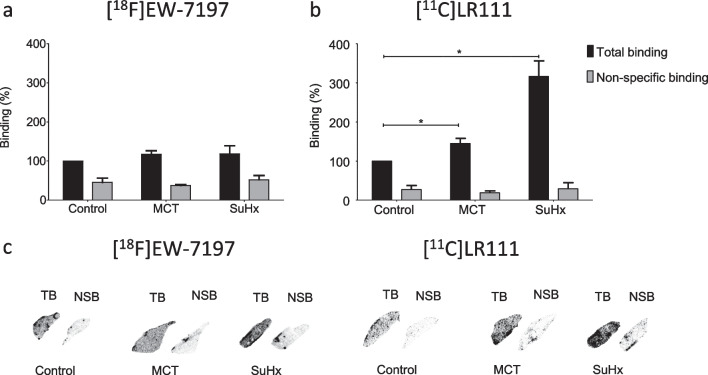
Autoradiography results: **a** per cent binding in control lungs (*n* = 3), MCT lungs (*n* = 3) and SuHx lungs (*n* = 3) for [^18^F]EW-7197; **b** per cent binding in control lungs (*n* = 3), MCT lungs (*n* = 3) and SuHx lungs (*n* = 3) for [^11^C]LR111; **c** Representative images of lung tissues for both tracers showing total binding (TB) and non-specific binding (NSB) in control lungs, MCT lungs and SuHx lungs. Error bars represent standard deviations. NSB images were obtained after blocking with SB431542. * represents a significant difference with SuHx lungs and MCT lungs (*p* < 0.05)

Binding of both tracers to ALK5 was investigated in healthy lung tissue (Wistar), MCT-exposed lungs (Wistar) and SuHx-exposed lungs (S.D.). Binding of [^18^F]EW-7197 to diseased lungs (both MCT- and SuHx-exposed lungs) showed 1.2 times higher binding compared with control lungs (Fig. 4a). Binding of [^11^C]LR111 to MCT-exposed and SuHx-exposed lungs was 1.5 and 3.2 times higher, respectively, than to control lungs (Fig. 4b). In addition, binding of both tracers decreased when SB431542 was added.

### Biodistribution and metabolite analysis in healthy Wistar rats

**Fig. 5:**
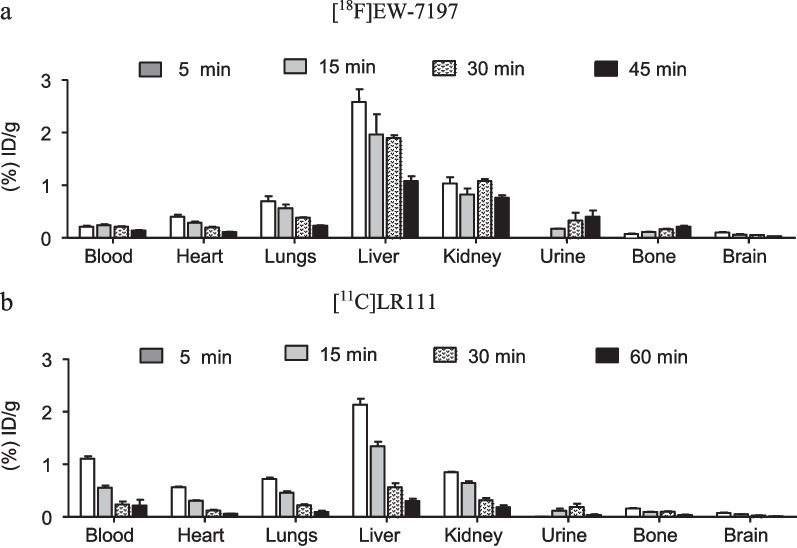
**a** [^18^F]EW-7197 and **b** [^11^C]LR111 uptake derived from ex vivo biodistribution studies. Tracers were administered to healthy male Wistar rats, which were sacrificed at 5, 15, 30, 45 or 60 min p.i. (*n* = 4 per time point). Results are expressed as injected dose per gram tissue. Error bars represent the standard error of the mean

**Fig. 6:**
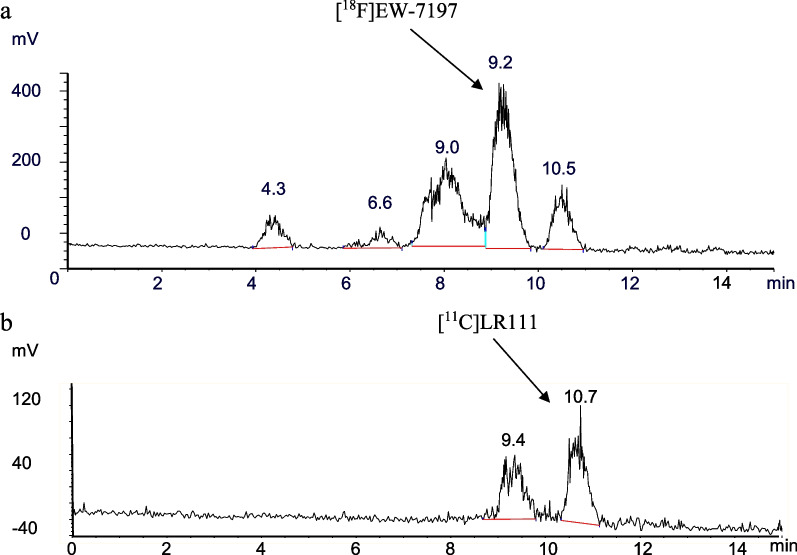
Radio-HPLC chromatograms of the non-polar metabolite fractions in rat plasma for **a** [^18^F]EW-7197 (*R*_t_ = 9.2 min) at 45 min p.i., and the non-polar metabolites (*R*_t_ = 4.3, 6.6, 9.0 and 10.5 min). **b** [^11^C]LR111 (*R*_t_ = 10.7 min) and the non-polar metabolite (*R*_t_ = 9.4 min) at 60 min p.i

Biodistribution data showed high liver and kidney uptake of both tracers (Fig. 5). Retention in bone (0.21 ± 0.02% injected dose per gram (ID·g^−1^)) was observed for [^18^F]EW-7197, indicating defluorination of the tracer. In addition, both tracers did not display high retention in healthy lung tissue, i.e. for [^11^C]LR111 0.45 ± 0.03% ID·g^−1^ at 15 min and for [^18^F]EW-7197 0.56 ± 0.07% ID·g^−1^ at 15 min (Fig. 5a, b). Metabolite analysis of [^18^F]EW-7197 showed 23 ± 5% intact tracer, 52 ± 1% polar metabolites and 25 ± 5% non-polar metabolites at 45 min p.i. (Table 2, Fig. 6a). [^11^C]LR111 showed 46 ± 2% intact tracer at 60 min p.i. (Table 2). At 60 min p.i., one major non-polar metabolite was formed (31 ± 0%) and multiple polar metabolites (23 ± 2%; Fig. 6b).

**Table 2:** Ex vivo metabolite analysis in blood of [^18^F]EW-7197 and [^11^C]LR111

Compound	[^18^F]EW-7197		[^11^C]LR111	
Time p.i. (min)	15	45	15	60
Intact tracer (%)	55 ± 16	23 ± 5	74 ± 5	46 ± 2
Non-polar metabolites (%)	17 ± 7	25 ± 5	21 ± 5	31 ± 0
Polar-metabolites (%)	28 ± 9	52 ± 1	5 ± 1	23 ± 2

### Echocardiography and haemodynamics

**Fig. 7:**
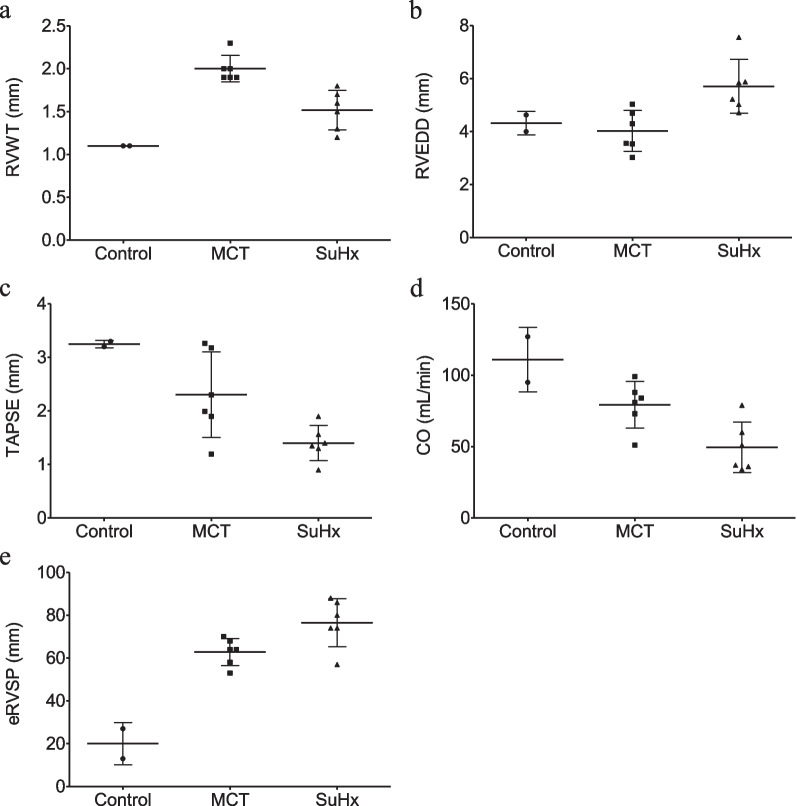
Echocardiographic parameters in control (*n* = 2), MCT-exposed (*n* = 6) and SuHx-exposed (*n* = 6) rats with **a** right ventricular wall thickness (RVWT), **b** right ventricular end diastolic diameter (RVEDD), **c** tricuspid annular plane systolic excursion (TAPSE), **d** cardiac output (CO) and** e** estimated right ventricular systolic pressure (eRVSP)

Right heart remodelling after MCT and SuHx exposure was confirmed by echocardiography (Fig. 7).

### PET study

**Fig. 8:**
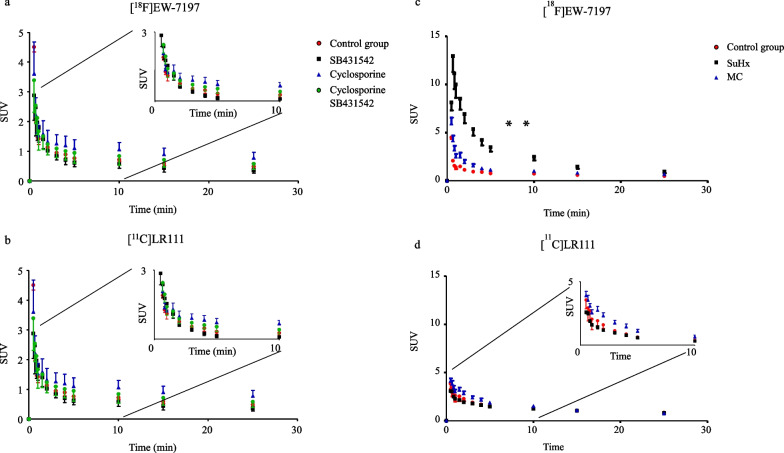
Time activity curves of [^18^F]EW-7197 (**a**) and [^11^C]LR111 (**b**) in healthy male Wistar rats (*n* = 4) Baseline conditions (red line), ALK5 blocked conditions (black line), efflux pump blocked conditions (blue line), efflux pump blocked and ALK5 blocked condition (green line). Time activity curves of [^18^F]EW-7197 (**c**) and [^11^C]LR111 (**d**) in healthy male Wistar rats (red line, *n* = 4), MCT-exposed rats (blue line, *n* = 6) and SuHx-exposed lungs (black line, *n* = 5) under baseline conditions

**Fig. 9:**
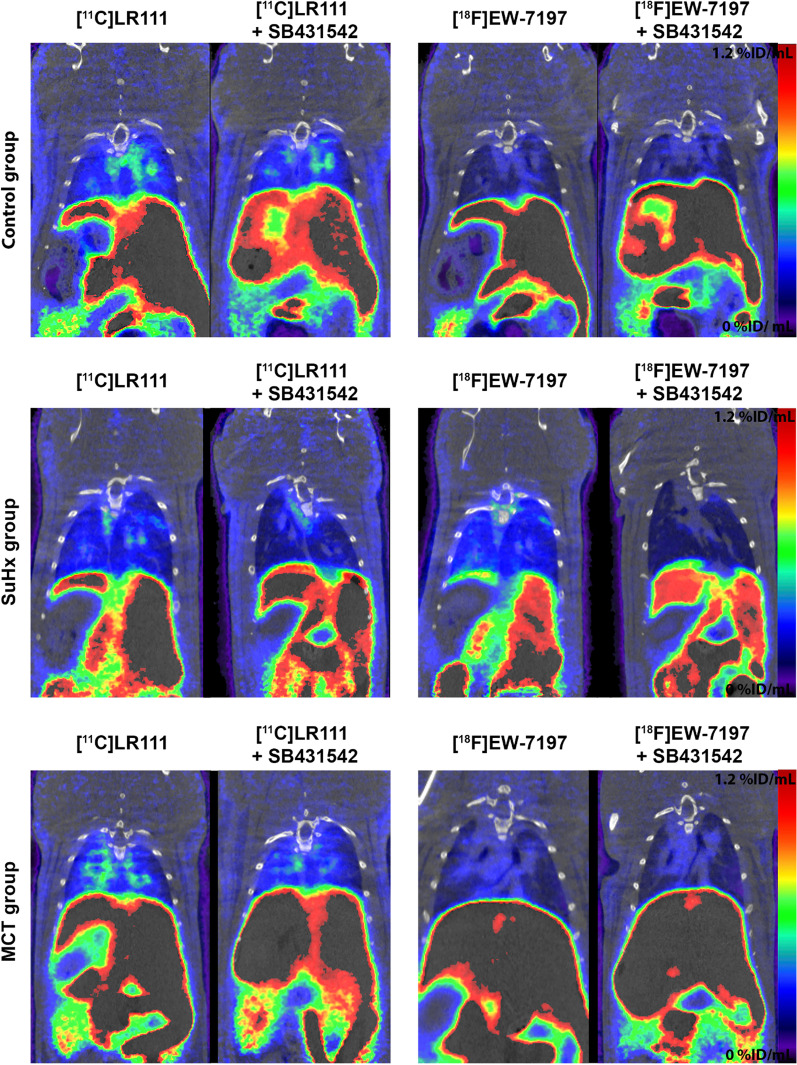
Representative PET images of [^18^F]EW-7197 and [^11^C]LR111 in healthy rats, SuHx rats and MCT rats with and without SB431542

In the dynamic PET imaging study in healthy male Wistar rats, the accumulation of [^18^F]EW-7197 was 1.5 fold higher in the lungs that received cyclosporine A (efflux pump inhibitor), 0.7 fold lower in the rats that received SB431542 (ALK5 inhibitor) and 1.2 fold higher in the rats that received cyclosporine A and SB431542 15 min p.i. (Table 3; Fig. 8a). For the dynamic PET imaging study with [^11^C]LR111 Cyclosporine A and SB431542 both caused a decrease in [^11^C]LR111 binding to the healthy lungs 0.9 and 0.7 fold respectively at 15 min p.i. (Table 3; Fig. 8b). The accumulation of [^18^F]EW-7197 in MCT-exposed lungs was 1.4 times higher compared with baseline conditions. In SuHx lungs, uptake was even higher (2.5-fold) compared with the control group at 15 min p.i. (Table 3; Fig. 8c). After 15 min, no difference in uptake was observed between the control group and diseased animals. Uptake of [^11^C]LR111 in the SuHx lungs was similar as uptake in control lungs. In MCT-exposed lungs, uptake was 1.1-fold higher than at baseline conditions (Table 3; Fig. 8d). After 15 min no difference in uptake was observed between the control group and the MCT-exposed rats. The PET scans of [^18^F]EW-7197 and [^11^C]LR111 showed high uptake in liver, kidneys and bladder (Fig. 9). [^18^F]EW-7197 showed more uptake in the diseased lungs than in the lungs of the healthy rats. In addition the uptake of the tracer could be blocked with SB431542 (Fig. 9). The PET scans of [^11^C]LR111 showed a lower uptake in the lungs of the SuHx rats than in the lungs of the control rats. The uptake of [^11^C]LR111 in the lungs of the MCT-exposed rats was higher than in the lungs of the control rats (Fig. 9). In two MCT-exposed rats high local uptake of [^18^F]EW-7197 and [^11^C]LR111 was observed in the lungs. The high uptake could be blocked with SB431542 (Additional file 1; Fig. 2).

**Table 3:** Uptake in the lungs (expressed as SUV) of [^18^F]EW-7197 and [^11^C]LR111 assessed by PET at 15 min p.i

	[^18^F]EW-7197	[^11^C]LR111
Baseline	0.59 ± 0.10	1.03 ± 0.07
Cyclosporine A	0.90 ± 0.18	0.85 ± 0.19
SB431542	0.44 ± 0.12	0.85 ± 0.12
Cyclosporine A + SB43142	0.72 ± 0.21	1.00 ± 0.32
MCT rats	0.81 ± 0.15	1.10 ± 0.25
SuHx rats	1.47 ± 0.47	1.04 ± 0.13

## Discussion

To investigate whether the ALK5 expression could be visualized in the lungs of PAH rat models, [^18^F]EW-7197 and [^11^C]LR111, two ALK5 targeting PET tracers, were evaluated in in vitro and in vivo PAH models.

The immunofluorescence data confirmed that ALK5 levels were increased in the lungs of MCT- and SuHx-exposed rats compared with control lungs. In addition, the apparent ALK5 expression was higher in the SuHx model than in the MCT model (Fig. 3a). In line with previous reports, ALK5 was predominantly observed in the endothelial and epithelial cells of the larger and smaller vessels of the pulmonary arteries [3–6]. Higher binding to diseased lungs than to control lungs, observed in the autoradiography study (Fig. 4), was in line with immunohistochemistry results, suggesting that both tracers are potential candidates for in vivo imaging of ALK5 in PAH rats.

The ex vivo biodistribution study showed fast clearance from the blood and rapid uptake in all organs (Fig. 5). The higher uptake in liver and kidneys can be explained by metabolism in the liver and excretion via the kidneys, respectively, and is considered normal for small molecules [28]. No substantial uptake of the tracers in healthy lungs was observed, which is an essential feature for measuring increased uptake in diseased lungs.

Analysis of in vivo metabolism of [^18^F]EW-7197 and [^11^C]LR111 in rats showed that both tracers are moderately stable. [^18^F]EW-7197 showed, next to multiple small fractions of non-polar metabolites, mainly polar metabolites (52 ± 1% after 45 min), which is favourable as polar metabolites will hardly affect the PET signal. [^11^C]LR111 showed two main non-polar metabolites and less polar metabolites (23 ± 2% after 60 min), which might explain the different behaviour of [^18^F]EW-7197 and [^11^C]LR111 in vivo. Unfortunately, it was not possible to determine the metabolic profile of both tracers in the lungs due to the low cell density and, consequently, low tracer uptake in the lungs.

As PAH displays the same hallmarks as cancer, MCT- and SuHx-exposed rats may have an increased efflux pump system [29]. Efflux pumps are mainly expressed on the apical surface of many epithelial and endothelial cells of the blood–brain barrier, but they are also known to be expressed in smaller numbers in lung and tumour tissues [30]. The role of these efflux pumps is to protect brain and lungs from foreign compounds and to transport small molecules back to the blood. Therefore, it was investigated whether [^18^F]EW-7197 and [^11^C]LR111 are substrates for P-gp, the dominant efflux transporter, to determine whether uptake of the tracers is not only a function of ALK5 expression, but also of P-gp.

Increased uptake of [^18^F]EW-7197 in the lungs of healthy rats was observed after administration of cyclosporine A and this uptake could be blocked by SB431542, an ALK5 inhibitor (Fig. 8a). This indicates that [^18^F]EW-7197 is a substrate for P-gp and, at the same time, a selective tracer for ALK5. The affinity of [^18^F]EW-7197 for P-gp may confound future studies in which binding of [^18^F]EW-7197 to ALK5 in the lungs will be assessed, as [^18^F]EW-7197 will be transported, at least in part, away from its target back into the blood before it can actually bind to ALK5.

In diseased animals, it was not possible to inject cyclosporine A because of the condition of the animals. Therefore, in these animals only [^18^F]EW-7197 and [^11^C]LR111 with and without SB431542 were injected. Uptake of [^18^F]EW-7197 was higher in both MCT- and SuHx-exposed rats than in controls, thereby showing the same pattern as the immunofluorescence data. This finding indicates that in MCT- and SuHx-exposed rats, [^18^F]EW-7197 can still detect ALK5 upregulation with PET imaging, even though P-gp might lower the uptake of [^18^F]EW-7197 in diseased lungs (Fig. 8c).

With [^11^C]LR111, different results were obtained. In healthy rats, the addition of cyclosporine caused a decreased tracer uptake in the lungs for unknown reasons, while SB431542 blocking also resulted in decreased tracer uptake in the lungs. In MCT animals, an increased uptake of [^11^C]LR111 in the lungs was observed compared with that in controls, but lung uptake in the SuHx animals was lower than in the control group. These mixed in vivo results indicate that [^11^C]LR111 would not be suitable for in vivo detection of ALK5 in rats, especially since these in vivo results did not correspond with in vitro results. A possible explanation is that one of the non-polar metabolites causes non-selective binding to ALK5 [31].

Several limitations of this (PET) study must be mentioned. First, the metabolite study of [^18^F]EW-7197 and [^11^C]LR111 in rats showed that both tracers are moderately stable whereby the [^11^C]LR111 is slightly more stable than [^18^F]EW-7197 (74 ± 5% 15 min p.i versus 55 ± 16% 15 min p.i). Therefore, the biodistribution study and de metabolite analysis was conduction 45 min p.i. for [^18^F]EW-7197 and 60 min p.i. for [^11^C]LR111.

Second, the MCT-exposure was performed in Wistar rats and the SuHx exposure was performed in Sprague Dawley rats. This because the MCT model develops better in Wistar rats and the SuHx model develops better in Sprague Dawley rats [17, 19]. The PET study included only Wistar rats as healthy control group whereby the healthy Wistar rats have a lower ALK5 expression than Sprague Dawley rats (Fig. 3a, b) in vitro*.* This might have as consequence that the difference in the in vivo PET imaging study would be less profound when compared with healthy Sprague Dawley rats. Third, no differences in the lungs between healthy controls and diseased animals were observed after 15 min. This fast clearance of the tracers in the lungs might give problems to diagnose PAH in the clinic without a careful planning and raises the question whether the tracer uptake in the lungs is due to a higher ALK5 expression or to changes in perfusion in the lungs of the diseased animals. This latter should be investigated in the further.

## Conclusion

[^18^F]EW-7197 and [^11^C]LR111 were assessed as potential PET tracers of ALK5 expression in two rat models of PAH. In vitro results clearly demonstrated ALK5 expression in both PAH models, confirming the usefulness of ALK5 as an imaging target for PAH. Both [^18^F]EW-7197 and [^11^C]LR111 showed selective binding in vitro. However, [^18^F]EW-7197 outperformed [^11^C]LR111 as an in vivo PET tracer of ALK5 and is therefore the preferred PET tracer to investigate further including in humans.

## Supplementary Information

**Additional file 1.** Supplementary Figures

## Data Availability

Experimental data are available from the corresponding author on reasonable request.
