# Supplementary material for: Imaging the TGFβ type I receptor in pulmonary arterial hypertension
Source: EJNMMI Res. 2023 Mar 22;13:23. doi: 10.1186/s13550-023-00966-7 (PMC10033812; doi:10.1186/s13550-023-00966-7)
Supplement: Supplementary file 1 — Additional file 1. Supplementary Figures [file 13550_2023_966_MOESM1_ESM.docx]

**Supplementary material: Imaging the TGFβ type I receptor in pulmonary arterial hypertension**

Lonneke Rotteveel^1^, Alex J. Poot^1^, Esther J.M. Kooijman^1^, Robert C. Schuit^1^, Ingrid Schalij^2^, Xiaoqing Sun^2^, Kondababu Kurakula^3^, Chris Happé^2^, Wissam Beaino^1^, Peter ten Dijke^3,4^, Adriaan A. Lammertsma^1^, Harm Jan Bogaard^2^, Albert D. Windhorst^1^

**Affiliations:**

^1^ Amsterdam UMC, VU University medical center, Dept. Radiology & Nuclear Medicine(s), (Amsterdam Cardiovascular Sciences), de Boelelaan 1117, Amsterdam, The Netherlands

^2^ Amsterdam UMC, VU University medical center, Dept. Pulmonary Medicine, (Amsterdam Cardiovascular Sciences), de Boelelaan 1117, Amsterdam, The Netherlands

^3^ Leiden University Medical Center, Dept. Cell and Chemical Biology, Einthovenweg 20, Leiden, The Netherlands

^4^ Oncode Institute and Leiden University Medical Center, Einthovenweg 20, Leiden, The Netherlands

Corresponding author: Lonneke Rotteveel, [l.rotteveel@amsterdamumc.nl](mailto:l.rotteveel@amsterdamumc.nl), Amsterdam UMC, VU University medical center, Dept. Radiology & Nuclear Medicine(s), (Amsterdam Cardiovascular Sciences), de Boelelaan 1117, Amsterdam, The Netherlands

Supplementary figure 1: The uncropped western blots. a) the uncropped gels belonging to figure 3 left panel b) the uncropped gels belonging to figure 3 right panel.

Supplementary figure 2: An example PET images with local uptake of [^18^F]EW-7197 and [^11^C]LR111 in SuHx-rats with and without SB431542.
